# Rapid and Sensitive Fluorescence Detection of *Staphylococcus aureus* Based on Polyethyleneimine-Enhanced Boronate Affinity Isolation

**DOI:** 10.3390/foods12071366

**Published:** 2023-03-23

**Authors:** Yujia Xu, Hongwei Zheng, Jianxin Sui, Hong Lin, Limin Cao

**Affiliations:** 1 Food Safety Laboratory, College of Food Science and Engineering, Ocean University of China, Qingdao 266100, China; 2 Key Laboratory of Marine Drugs, The Ministry of Education of China, School of Medicine and Pharmacy, Institute of Evolution & Marine Biodiversity, Ocean University of China, Qingdao 266100, China

**Keywords:** boronate affinity, *Staphylococcus aureus*, enrichment, fluorescent probes

## Abstract

There are increasing demands for fast and simple detection of pathogens in foodstuffs. Fluorescence analysis has demonstrated significant advantages for easy operation and high sensitivity, although it is usually hindered by a complex matrix, low bacterial abundance, and long-term bacterial enrichment. Effective enrichment procedures are required to meet the requirements for food detection. Here, boronate-functionalized cellulose filter paper and specific fluorescent probes were combined. An integrated approach for the enrichment of detection of *Staphylococcus aureus* was proposed. The modification of polyethyleneimine demonstrated a significant effect in enhancing the bacterial enrichment, and the boronate affinity efficiency of the paper was increased by about 51~132%. With optimized conditions, the adsorption efficiency for *S. aureus* was evaluated as 1.87 × 10^8^ CFU/cm^2^, the linear range of the fluorescent analysis was 10^4^ CFU/mL~10^8^ CFU/mL (R^2^ = 0.9835), and the lowest limit of detection (LOD) was calculated as 2.24 × 10^2^ CFU/mL. Such efficiency was validated with milk and yogurt samples. These results indicated that the material had a high enrichment capacity, simple operation, and high substrate tolerance, which had the promising potential to be the established method for the fast detection of food pathogens.

## 1. Introduction

In recent years, foodborne pathogenic-bacteria-related events have frequently occurred worldwide and significantly impacted socio-economic development [1]. Common foodborne pathogenic bacteria include *Listeria monocytogenes*, *Escherichia coli*, *Salmonella* spp., and *Bacillus cereus*, all of which can cause adverse health effects. *Staphylococcus aureus* could cause a range of life-threatening complications in humans, from minor infections to fatal sepsis. It is considered one of the most critical pathogens in dairy products, aquatic products, and other foodstuffs [2,3]. For effective bacterial control in food chains, there is now a great demand for accurate and rapid analytical techniques to replace traditional plate counting methods, which are both time and labor consuming [4]. For this purpose, many novel strategies have been developed, such as various polymerase chain reaction (PCR) tests, immunoassays, and biosensors [5,6,7]. In recent years, fluorescence analysis has been gaining increasing attention due to its powerful simplicity and rapidity compared with other techniques [8]. With the development of aggregation-induced emission, quantum dots, composite fluorescent probes, and other new techniques, the specificity and sensitivity of fluorescence analysis could also be significantly improved [9,10]. However, in many cases the complex food matrix components could significantly interfere with fluorescent probes, making it very difficult to capture low-content bacteria and greatly limiting the real detection of foodstuffs [11]. Traditionally exploited pretreatments, such as filtration, centrifugation, and magnetic separation, in many cases could not fulfill the requirements for poor enrichment efficiency, high cost, and low capacity [12]. Therefore, more effective enrichment and isolation of target bacteria from complex matrices seem of great importance for further development of these techniques.

Boric acid groups can covalently conjugate with cis-dihydroxyl compounds to form reversible and pH-controlled cyclic esters [13]. Considering the rich content of cis-dihydroxyl compounds on the surface of bacteria walls, such as lipopolysaccharide, peptidoglycan, and glycoprotein, recently boronate affinity has been reported as a new and effective tool for selective separation of bacteria in surface-enhanced Raman scattering and electrochemical analysis [14,15]. Various carriers such as agarose, cryogels, and magnetic nanobeads have been used to prepare boronate affinity materials [16,17,18] and demonstrated the potential to fulfill certain requirements. Until now, the effective combination of boronate affinity with fluorescent assays for food pathogens was still very limited. In most previous studies, the boronate affinity groups were directly coupled with fluorescent probes [19,20] but this proved difficult to reach satisfactory specificity and sensitivity in biological matrices, and some of them can only perform semi-quantitative detection. Insufficiency in boronate groups was considered one of the main reasons [21]. This may result in not only poor enrichment of target bacteria but also significant interference induced by the non-specific action with matrix components [22,23]. In order to improve the efficiency and simplicity of fluorescence detection in food, we considered the preparation of a thin-film boronate affinity material with high enrichment capacity. The boronate affinity material was then combined with fluorescence in situ hybridization. Thus, an integrated detection method from enrichment to detection of pathogenic bacteria could be constructed.

Herein, we chose cellulose filter paper as the substrate to fabricate boronate affinity materials because of its low cost, hydrophilic properties, biocompatibility, ease of modification, and abundance of active surface sites [24,25]. To increase the density of 3,5-difluoro-4-formylphenylboronic acid (DFFPBA) and hence increase the affinity efficiency to the target bacterium, polyethyleneimine (PEI) was introduced onto the surface of the paper to construct a branched conformation. Then, a boronate affinity filter-paper-based fluorescent analysis was established for *S. aureus* and its efficiency was evaluated and validated. The results allowed us to suggest it as a new and promising technique for fast detection of the pathogen in foodstuffs.

## 2. Materials and Methods

### 2.1. Reagents

3,5-Difluoro-4-formyl-phenylboronic acid (DFFPBA) was purchased from Shanghai Yuanye Biotechnology Co. Polyethyleneimine (PEI, Mw = 10,000), formamide, and sodium cyanoborohydride (NaBH_3_CN) were purchased from Shanghai Macklin Biochemical Co. Sodium dodecyl sulfate (SDS), tris (hydroxymethyl) aminomethane (Tris-basic), paraformaldehyde, and lysozyme were purchased from Solarbio (Beijing, China). Epichlorohydrin, sodium borohydride (NaBH_4_), sodium hydroxide (NaOH), sodium bicarbonate (NaHCO_3_), sodium chloride (NaCl), sodium carbonate (Na_2_CO_3_), sodium acetate, ammonium molybdate, acetic acid, methanol, ethanol, acetonitrile (ACN), and fructose were purchased from China Pharmaceutical Group Corporation. The cellulose filter paper was purchased from Fushun Civil Affairs Filter Paper Factory (No.102, with a diameter of 70 mm, an average pore size of 11 μm, and a thickness of 0.2 mm). Milk and yogurt were produced by Mengniu Dairy Co., Ltd., Hohhot, China. The fluorescent probe specific for *S. aureus* was synthesized by Qingdao Tsingke Biotechnology Co., Ltd., Qingdao, China. according to previous studies [26]; the sequence was 5′-GAGCGTGCTGGATTTGTGT-3, modified with carbonyl fluorescein carboxyfluorescein (FAM) at the 5’ end. FAM has an excitation wavelength of 493 nm and an emission wavelength of 518 nm, emitting yellow-green fluorescence under the fluorescence microscope. The specificity of the fluorescent probes was investigated referring to the method in the literature [27]. Fluorescent in situ hybridization of several different bacteria (*S. aureus*, *Salmonella enterica serovar Typhimurium*, *Escherichia coli*, *Vibrio parahaemolyticus*, and *Listeria monocytogenes*) was performed using specific probes and the non-specific DNA dye DAPI. DAPI has an excitation wavelength of 358 nm and an emission wavelength of 461 nm, emitting blue fluorescence under the fluorescence microscope. The results were observed under a laser confocal microscope.

### 2.2. Apparatus

Fourier transform infrared (FT-IR) spectroscopy was performed on a Fourier transform infrared spectrometer (Thermo Fisher Scientific, Nicolet iS10, Waltham, MA, USA). Fluorescence intensity was measured on a fluorescence spectrophotometer (Hitachi, F-4600, Tokyo, Japan). Fluorescence images of bacteria were observed using a laser confocal scanning microscope (Nikon, A1R HD25, Tokyo, Japan).

### 2.3. Bacterial Strains and Culture Conditions

*S. aureus* ATCC 6538, *Salmonella enterica serovar Typhimurium* ATCC 14028, *Escherichia coli* ATCC 25922, *Vibrio parahaemolyticus* ATCC 17802, and *Listeria monocytogenes* ATCC 19115 were from the Food Safety Laboratory, Ocean University of China. Strains were cultured in lysogeny broth (LB) liquid medium. Individual colonies were picked and incubated in 10 mL tubes containing 5 mL of LB liquid medium at 37 °C for 18 h with 180 r/min oscillation. The bacteria strains were collected by centrifugation at 7000× *g* for 2 min, washed three times with phosphate-buffered saline (PBS, 0.01 M, pH 7.4), and re-suspended in 1 mL of PBS. The optical density was then measured at 600 nm.

### 2.4. Preparation of Boronate Affinity Paper@epoxy@PEI-DFFPBA

The boronate affinity filter paper was prepared by reference to the method in the literature [16] with slight modifications. First, the cellulose filter paper was soaked in methanol for 15 min to remove impurities and dried naturally. Then, the epoxy-modification process of the paper was prepared by epichlorohydrin activation with slight modifications. A total of 1 g of the filter paper was immersed in a mixture of epichlorohydrin (8 mL), NaBH_4_ (0.8 g/L, 10 mL), and NaOH (0.9 M, 10 mL) with stirring at 40 °C for 4 h. After that, the paper was thoroughly washed with deionized water. Next, 1 g of the epoxy-modified filter paper was added to a mixture of Na_2_CO_3_ (50 mM, 50 mL) and PEI (1 g) and stirred at room temperature for 12 h. The grafted filter paper was subsequently rinsed with Na_2_CO_3_ and distilled water and then dried. A total of 0.2 g of the amino-modified filter papers was treated with a mixture of DFFPBA, NaBH_3_CN, ACN (10 mL), and methanol (5 mL) for 72 h at room temperature. The ligand DFFPBA was modified onto paper through Schiff-base reaction. Finally, the obtained paper@epoxy@PEI-DFFPBA was washed in turn with methanol, 5% NaHCO_3_, 5% NaCl, and deionized water to remove unreacted residues. The chemical composition of the amino-modified and boronate affinity filter papers was examined by Fourier transform infrared spectroscopy (FTIR).

In order to investigate the optimal amount of phenylboronic acid addition, the addition of DFFPBA was set to 500 mg, 750 mg, 1000 mg, 1250 mg, and 1500 mg per 1 g of paper@epoxy@PEI corresponding to 1000 mg, 1500 mg, 2000 mg, 2500 mg, and 3000 mg of NaBH_3_CN, respectively. The adsorption efficiency of paper@epoxy@PEI-DFFPBA with different phenylboronic acid additions on tea polyphenols was investigated following the method in the literature [28]. The amount of tea polyphenol absorbed by the boronate affinity filter paper was determined as follows: one piece of prepared paper@epoxy@PEI-DFFPBA was mixed with tea polyphenol standard solution (1.0 mg/mL, 2 mL). After incubating at 37 °C for 15 min, 0.2 mL of the solution was collected and ammonium molybdate solution (100 g/L, 0.1 mL) and HAc-NaAc buffer (pH 3.5, 2.2 mL) was added. A total of 200 μL of the mixed solution was added to a 96-well plate, and the absorbance was measured at 341 nm. The concentration was calculated from the absorbance before and after enrichment, and then, the adsorption efficiency was calculated. The adsorption efficiency (A) was calculated as follows:

(1) $$A=\frac{c_{0}-c_{1}}{c_{0}}$$

where C_0_ (mg/mL) is the initial concentration of tea polyphenols and C_1_ (mg/mL) is the concentration of tea polyphenols in the solution after enrichment.

Meanwhile, boronate affinity filter paper without PEI was prepared: the epoxy-modified filter paper was added to a 1:1 (*v*/*v*) mixture of ammonia and acetonitrile for 12 h at 60 °C. The rest of the steps were the same as the preparation of the boronate affinity filter paper above. The effect of the introduction of PEI on the adsorption efficiency of boronate affinity filter paper was investigated with tea polyphenols using *S. Typhimurium* and *S. aureus* as the targets.

### 2.5. Isolation of Paper@epoxy@PEI-DFFPBA to S. aureus

Referring to the previous method [16], the boronate affinity filter paper was immersed in PBS buffer (0.01 M, pH 7.4) containing *S. aureus* and incubated at 37 °C for several minutes with 180 r/min shaking. After that, the filter paper was removed, washed with 2 mL of PBS buffer, and eluted with 1 mL of eluent. 

The bacterial concentrations in the solution before and after enrichment, as well as in the washing solution were determined using the conventional plate smear counting method. The adsorption efficiency was calculated in the same way as (1). The bacteria adsorption capacity (Q) and elution efficiency (E) was calculated by the following equation.

(2) $$Q=\frac{(c_{0}-c_{1})v_{0}-c_{2}v_{2}}{S}$$

(3) $$E=\frac{c_{3}v_{3}}{(c_{0}-c_{1})v_{0}-c_{2}v_{2}}$$

where C_0_ (CFU/mL) is the initial concentration of bacterial suspension and C_1_ (CFU/mL) and C_2_ (CFU/mL) are the concentrations of bacteria in the bacterial suspension and washing solution after enrichment, respectively. C_3_ (CFU/mL) is the bacterial concentration of eluate; V_0_ (mL) and V_1_ (mL) are the volumes of bacterial suspension and washing solution, respectively; V_2_ is the volume of eluent; and S (cm^2^) is the area of boronate affinity filter paper.

### 2.6. Fluorescent Detection of S. aureus

Paper@epoxy@PEI-DFFPBA absorbed the bacteria under optimized conditions. Then, the paper was placed on a slide and dried at room temperature. A total of 20 μL of 1 mg/mL lysozyme solution (10 mM Tris-basic, pH 7.5) was added dropwise to paper@epoxy@PEI-DFFPBA and the paper was incubated in a wet box at 37 °C for 10 min before being rinsed with deionized water. A total of 20 μL of lysostaphin solution (10 mM Tris-basic, 10 μg/mL, pH 7.5) was added dropwise to paper@epoxy@PEI-DFFPBA, and then, the paper was incubated at 37 °C for 10 min before being rinsed with deionized water. Probes targeting the Fem B gene of *S. aureus* was used for hybridization. A total of 50 μL of 10 ng/mL probe solution (20 mM Tris-HCl pH 7.2, 0.9 M NaCl, 1% SDS, 20% formamide) was added dropwise to the filter paper, and it was incubated in the dark at 50 °C for 150 min. The filter paper was sequentially washed with 1 mL of washing buffer (48 °C, 20 mM Tris-HCl pH 7.2, 0.18 M NaCl) and 1 mL of water before being eluted with 1 mL of 0.5 M fructose for 10 min. Finally, the fluorescence emission spectra of the washing buffer and eluent were measured at an excitation wavelength of 493 nm. The fluorescence peak at 518 nm was extracted, plotted against the bacterial concentration’s common logarithm (log), and fitted to a linear model. The limit of quantification (LOQ) and the limit of detection (LOD), as defined by the International Union of Pure and Applied Chemistry (IUPAC), were calculated as follows: the response value corresponding to the LOD is the mean value of the blank plus 3 times its standard deviation and the LOQ is 3.3 times the LOD.

### 2.7. Validation in Food Matrix

Milk samples were spiked with *S. aureus* to achieve a concentration range of 10^4^~10^7^ CFU/mL. A total of 1 mL of the milk sample was adsorbed by 1 cm^2^ paper@epoxy@PEI-DFFPBA for 30 min at 37 °C. After washing with PBS (0.01 M, pH 7.4), the samples before and after enrichment were plate coated and counted to detect changes in bacterial concentration. A total of 1 cm^2^ of adsorbed paper@epoxy@PEI-DFFPBA was treated with 10 μL 1 mg/mL lysozyme and 10 μL 10 μg/mL lysostaphin sequentially for 10 min at 37 °C and then washed with water. Next, 50 μL 10 ng/mL of fluorescent probe solution was added, and this was reacted in the dark at 50 °C for 150 min. Afterward, the filter paper was washed with 1 mL of washing buffer (48 °C, 20 mM Tris-HCl pH 7.2, 0.18 M NaCl). Finally, the bacteria were eluted for 10 min with 1 mL of 0.5 M fructose, and the concentration of bacteria in the eluate was measured by fluorescence, as previously mentioned.

### 2.8. Statistical Analysis

All coating and testing were performed in triplicate. The mean ± standard deviation (SD) was used to represent numerical variables with normal distributions. Student’s *T* test and the ANOVA test were used for statistical analysis to reveal any significant differences in the adsorption capacity, adsorption efficiency, and fluorescence intensity of the borate-affinity filter paper under different conditions. Student’s *T* test was used to compare the differences between the two groups of data, and the ANOVA test was used to compare three or more groups of data. *p* values < 0.05 were considered statistically significant. Graphs were prepared in Graphpad Prism (version 8.0.2).

## 3. Results

### 3.1. The Effect of Polyethyleneimine Modification to Improve the Boronate Affinity Ability of the Filter Paper

**Figure 1:**
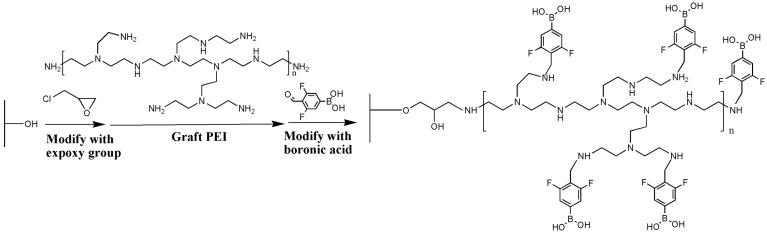
The synthesis scheme of paper@epoxy@PEI-DFFPBA.

In our previous studies, PEI demonstrated satisfactory efficiency as the scaffold for the amplification of ligand density on the surface of sepharose gels [16,29]. Therefore, a similar strategy was proposed to enhance the coupling efficiency of boronic groups on the filter paper (Figure 1).

**Figure 2:**
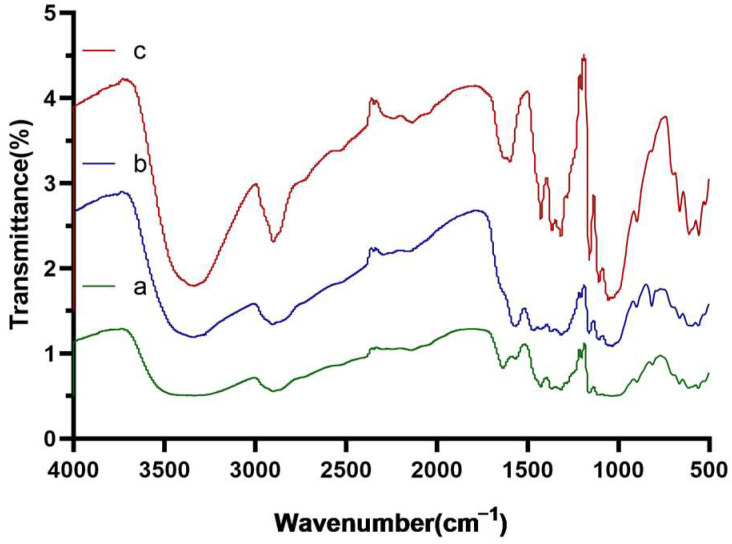
Fourier infrared spectra of the prepared paper@epoxy@PEI-DFFPBA (a), paper@epoxy@PEI (b), and unmodified cellulose filter paper (c).

**Figure 3:**
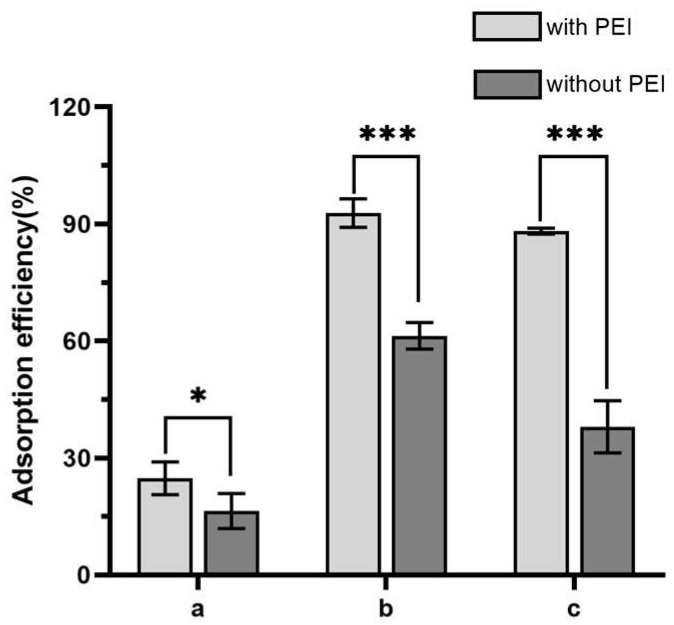
The adsorption efficiency of paper@epoxy@PEI-DFFPBA to tea polyphenol (a), *Salmonella Typhimurium* (b), and *Staphylococcus aureus* (c) with and without PEI modification. Bars show the means and the error bars show standard deviations. Asterisks indicate significant difference levels, * *p* < 0.05, *** *p* < 0.001.

The success of the modification was confirmed by the Fourier infrared spectra (Figure 2). The absorption peaks at 3338 cm^−1^ and 3278 cm^−1^ (typical for -NH_2_ groups) indicated the effective introduction of PEI. Successful immobilization of DFFPBA to paper@epoxy@PEI was confirmed by the skeleton vibration of benzene ring at 1635 cm^−1^ and 1427 cm^−1^ [30]. When tea polyphenol was used as the representative cis-dihydroxy compound, the absorption efficiency of the filter paper increased by around 51% (Figure 3) compared with that without the introduction of PEI. For *S. Typhimurium* and *S. aureus*, the adsorption efficiency increased by 51% and 132%, respectively. The rich content of amine in PEI may not only enable the ring-opening reaction between PEI and epoxy-modified filter papers but also provide more active sites to conjugate with the carboxyl groups of DFFPBA [10], which could significantly increased the boronic group density and the efficiency of capturing cis-dihydroxyl compounds.

**Figure 4:**
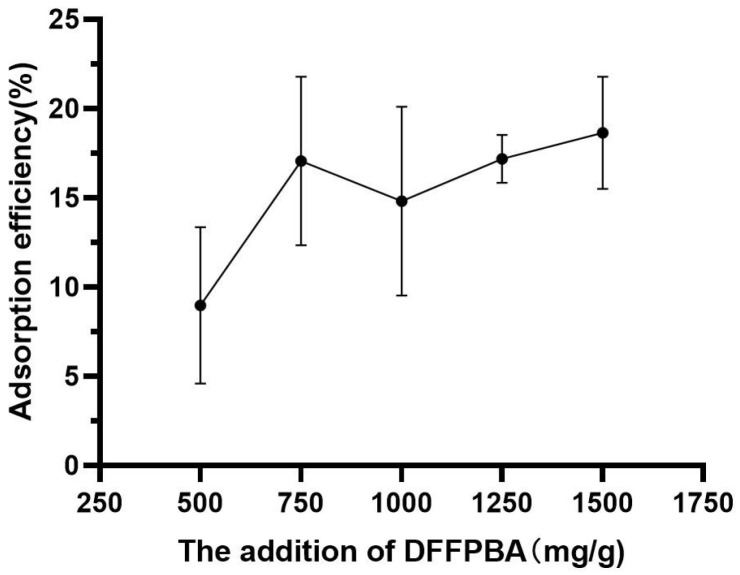
The effect of DFPBA content on the absorption efficiency of prepared paper@epoxy@PEI-DFFPBA to tea polyphenol. The error bars show standard deviations.

The DFFPBA addition demonstrated a significant influence on PEI-introduced modification. Within the range investigated, the DFFPBA usage of 750 mg DFFBPA/g (filter paper) was observed as the optimal (Figure 4). With such content, the adsorption efficiency of tea polyphenol was much higher than that of 500 mg DFFBPA/g (filter paper). However, no significant difference was demonstrated with a further increase in DFFPBA (*p* > 0.05). 

### 3.2. Optimization of Analytical Performance

**Figure 5:**
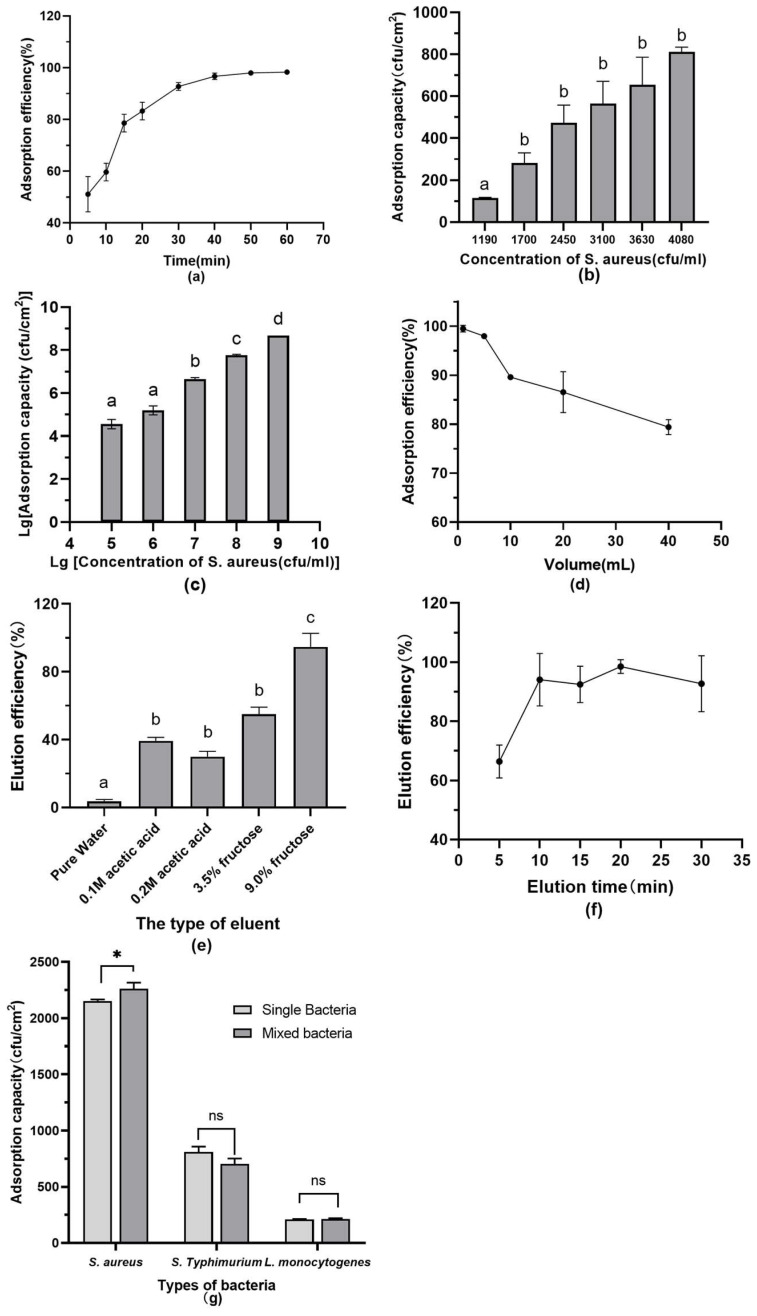
Effect of enrichment time (**a**), low bacterial concentration (**b**), high bacterial concentration (**c**), volume (**d**), eluent type (**e**), elution time (**f**), and mixed bacteria (**g**) on the adsorption efficiency or capacity of *S. aureus* by paper@epoxy@PEI-DFFPBA. Absorption capacity without bacteria is 0. Bars show the means and the error bars show standard deviations. The results of ANOVA analysis showed significant differences in Figure 5b,c,e. Asterisks indicate significant difference levels; * *p* < 0.05, ns *p* > 0.05. The a, b, c, d in the figure indicate significant differences between multiple groups.

The effect of time, volume, and other parameters on the boronate affinity isolation of *S. aureus* were investigated, and the results were summarized in Figure 5. The filter paper showed a significant time-dependent enrichment to the bacteria. This was consistent with the recognized principle for boronate affinity with cis-dihydroxy compounds [31]. Here, the absorption efficiency dramatically increased up to 92.73% at the 30th minute of the interaction and remained almost constant. 

At the *S. aureus* concentration of 2 × 10^9^ CFU/mL, the absorption capability reached 1.87 × 10^8^ CFU/cm^2^ (2.40 × 10^7^ CFU/mg), approximately 2.6~112 times that of other boronate affinity materials [17,18]. Moreover, unlike many previous studies in which the adsorption efficiency was mainly evaluated with high concentrations [16,17,18], there was still satisfactory adsorption efficiency over 96.70% at *S. aureus* concentrations lower than 10^4^ CFU/mL. This would be of great importance in real fast detection of bacteria by PCR, fluorescence, and other techniques, which usually have a sensitivity of 10^3^ CFU/mL~10^4^ CFU/mL. Such a significantly enhanced boronate affinity efficiency may be attributed to the large specific surface area given by the cellulose filter paper’s three-dimensional mesh and porous structure [32], as well as the multi-locus synergistic effect provided by fluorophenylboronic acid and hyperbranched polyethyleneimine.

In principle, the dissociation of boronate-cis-diol compounds was easily conducted under acidic conditions. Competitive elution by fructose or other cis-diol compounds with stronger affinity was also suggested as an effective method [33,34]. Here, the elution efficiency with pure water, 0.1 M acetic acid, 0.2 M acetic acid, 3.5% fructose, and 9.0% fructose were investigated, and 9.0% fructose was observed as the best result (*p* < 0.05). With an elution time of 10 min, 94.44% of the absorbed bacteria could be eluted from paper@epoxy@PEI-DFFPBA. In addition, such a competitive elution could effectively prevent the possible death of bacteria in an acidic environment and subsequent loss or degradation of nucleic acids [35]. This would be of significance to improve the sensitivity and accuracy of the following fluorescent analysis. 

The results in the mixed bacterial solution showed that the adsorption capacity of *S. aureus* was not affected by other bacteria in the mixed solution. The surface polysaccharides of *S. aureus* contained many more cis-dihydroxy structures than those of other strains. In addition, surface polysaccharides of *S. aureus* consisted of N-acetylmuramic acid and N-acetylglucosamine linked alternately by β-1,4 linkages, further accentuating a large number of cis-dihydroxy structures. The phenylboronic acid material in the previous study [36] also showed a much higher adsorption capacity for *S. aureus* than other strains.

**Figure 6:**
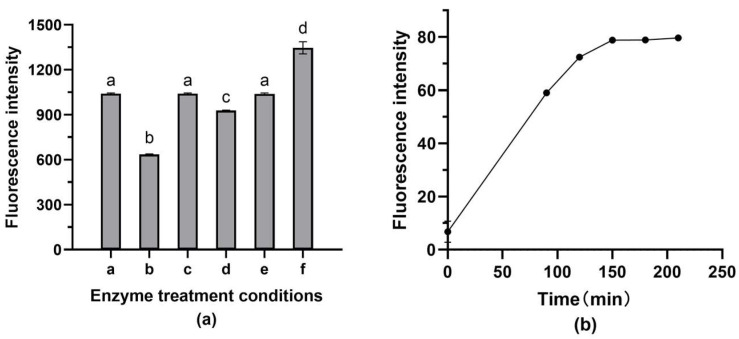
Effects of enzyme treatment conditions on the fluorescent detection of *S. aureus*. (**a**) a: 1 mg/mL lysozyme; b: 1 mg/mL lysozyme and 10 μL/mg lysostaphin; c: 2 mg/mL lysozyme and 10 μL/mg lysostaphin; d: 5 mg/mL lysozyme and 10 μL/mg lysostaphin; e: 10 μL/mg lysostaphin; and f: treatment without the addition of enzymes. (**b**) Hybridization time. Bars show the means and the error bars show standard deviations. The a, b, c, d in the figure indicate significant differences between multiple groups.

For the fluorescent assay of isolated *S. aureus*, enzymatic treatments demonstrated a significant influence on the probe–bacteria hybridization on paper@epoxy@PEI-DFFPBA (Figure 6). With 1 mg/mL lysozyme and 10 μg/mL lysostaphin to react for 10 min, the fluorescence intensity in the washing buffer was the lowest (*p* < 0.05), indicating that most fluorescent probes were bound to bacterial DNA. Moreover, 2.5 h seemed long enough for the hybridization, and the fluorescence intensity in the reaction solutions demonstrated no further significant increase after then (*p* > 0.05).

### 3.3. Evaluation of the Analytical Efficiency

**Figure 7:**
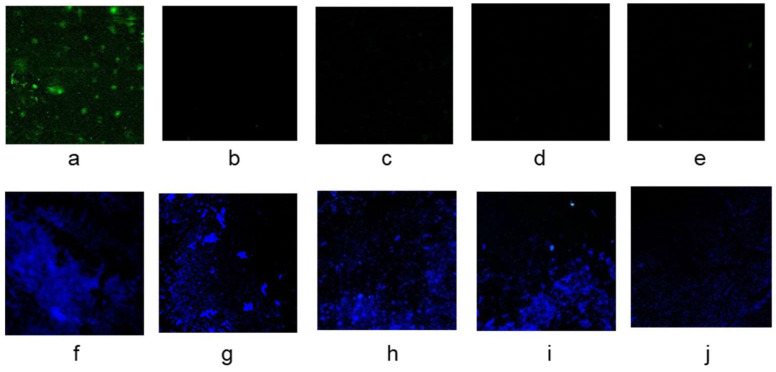
Fluorescence microscopy results after in situ hybridization of the specific probes with *S. aureus* (**a**), *S. Typhimurium* (**b**), *L. monocytogenes* (**c**), *E. coli* (**d**), and *V. parahaemolyticus* (**e**) followed by DAPI staining (**f–j**).

A fluorescent probe targeting the *S. aureus* Fem B gene was used in the study. This is a highly conserved and specific chromatin marker that distinguishes *S. aureus* from other *staphylococci*. The specificity of the method was evaluated by the in situ hybridization of the fluorescent probe with *S. aureus*, *S. Typhimurium*, *L. monocytogenes*, *E. coli*, and *V. parahaemolyticus*, which are common pathogens in foodstuffs and represented both Gram-negative and Gram-positive bacteria. As shown in Figure 7, significant yellow-green fluorescence could only be observed after hybridization with *S. aureus* and indicated satisfactory specificity of the analysis.

**Figure 8:**
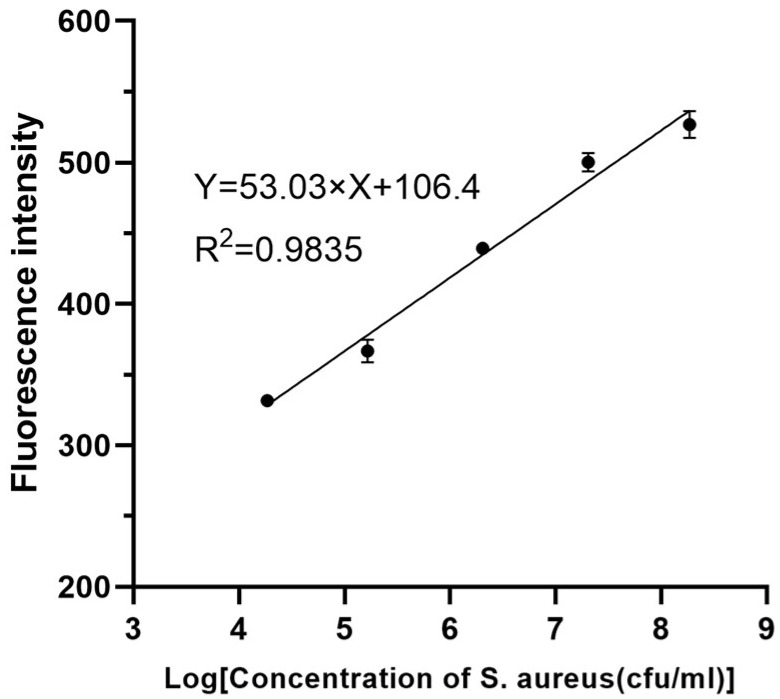
Plot of fluorescence intensity versus log of bacterial concentration in PBS.

The efficiency of the boronate affinity fluorescent assay was investigated under the optimized conditions. With the concentration of *S. aureus* ranging from 10^4^ CFU/mL~10^8^ CFU/mL in PBS, there was a significant linear relationship between fluorescence intensity and the bacterial concentration. The linear regression equation was expressed as y = 52.03x + 106.4, with a correlation coefficient (R^2^) of 0.9835 (Figure 8). The LOQ and LOD of the established method were calculated as 7.39 × 10^2^ CFU/mL and 2.24 × 10^2^ CFU/mL, respectively. The whole test (including pre-isolation of the bacteria) could be completed in 3.5 h. 

**Figure 9:**
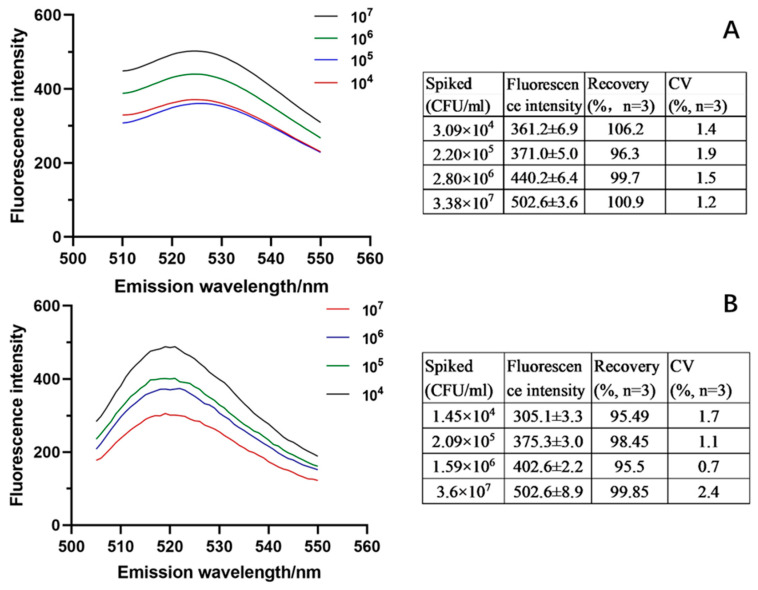
Results of paper@epoxy@PEI-DFFPBA-based fluorescence analysis of *S. aureus* in milk (**A**) and yogurt (**B**) samples.

The real efficiency of the established technique was further validated with milk and yogurt samples. The adsorption efficiency was evaluated as 83.67%, about 88.60% of that in PBS, which confirmed excellent tolerance of the material to food matrices. With the spiking concentration of 10^4^ CFU/mL~10^7^ CFU/mL, the recovery was calculated as 95.49~106.2% and the CV was 0.7~2.4% (Figure 9). Although the yogurt contains a large number of *Lactobacillus*, the method still showed good detection results. All these results verified the satisfactory accuracy and precision of the technique in complex foodstuffs.

## 4. Discussion

**Figure 10:**
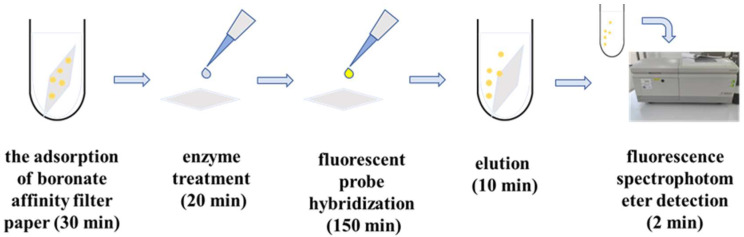
Flow chart of the process of enrichment to detection of pathogenic bacteria.

In this study, a boronate affinity cellulose filter paper with high affinity, high adsorption capacity, and excellent substrate tolerance was prepared. The boronate affinity material was used in combination with the fluorescence in situ hybridization technique to construct an integrated rapid detection strategy for pathogenic bacteria from enrichment to detection (Figure 10). This new assay could open up new ideas for the rapid detection of foodborne pathogenic bacteria.

Various fluorescent analyses have been developed for fast detection of food pathogens, such as real-time fluorescence PCR [37], fluorescent in situ hybridization (FISH) [38], fluorescent nano-sensors [10], etc., (Table 1). However, the real application of these techniques seems difficult, which resulted in significantly decreased rapidity and simplicity of the whole performance. For example, q-PCR and FISH require pre-enrichment cultures of samples, which often take 5–8 h. Current research combining boronate affinity with fluorescence detection has shortened the detection times but has less application in food matrices. Here, we proposed an integrated detection strategy for *S. aureus* by combining cellulose-filter-paper-based boronate affinity isolation and subsequent fluorescent hybridization. Compared with q-PCR methods, this method does not require enrichment culture, thus saving time. The whole process includes the adsorption of boronate affinity filter paper (30 min), enzyme treatment (20 min), fluorescent probe hybridization (150 min), elution (10 min), and fluorescence spectrophotometer detection (2 min). In addition, the test requires only a thermostatic incubator with a fluorescence spectrophotometer, which does not require complex operations or equipment. The cost per sample may be a few dollars or even less, considering the reuse of the eluted boronate affinity material. Compared with detecting bacteria by direct connection of phenylboronic acid and fluorescent probes, this method has lower detection limits and could meet the requirements for detecting *S. aureus* in food. The established technique demonstrated excellent tolerance to the complex food matrix, and satisfactory accuracy, sensitivity, precision, and rapidity were demonstrated in milk samples. 

The observed achievement may be due to many reasons, such as the following: (i) In previous studies, the small pore size of some boronate affinity materials [40] led to the difficulty of the rapid passage of bacteria, making them unable to take full advantage of the high specific surface area. It is prone to clogging when applied to complex matrices such as aquatic products [16]. In contrast, the large-pore-size filter paper was less likely to cause clogging of tiny particles such as proteins or surface adsorbed substances [41]. In addition, magnetic particles are easily lost during elution recovery [18]. The formed filter paper does not have this problem and can be cut to meet different experimental needs. It is suitable for processing of large sample volumes [42,43]. (ii) The successful modification of PEI greatly enhanced the density of boronic groups on the filter paper surface, leading to a much-improved boronate affinity ability to enrich target bacteria more easily and rapidly [44]. Moreover, according to previous studies [45,46], the chosen borate affinity group, fluorophenylboronic acid, is able to adsorb bacteria more rapidly and efficiently in lower pH physiological environments due to its low pK(a). (iii) Boronate affinity isolation and nucleic acid probes provided dual specificity of binding. They prevented the assay from being easily interfered with by coexisting components. Enrichment before detection can reduce false positive results due to the non-specific binding of the fluorescent probe to substances in the matrix during the assay [47]. Such a multi-locus synergy would be helpful in improving the sensitivity and accuracy of the detection. In summary, boronate affinity cellulose filter paper was designed and prepared using the multi-locus synergy conferred by hyperbranched polymers. A fluorescent in situ detection method for *S. aureus* based on boronate affinity materials was constructed. It provides a reference for the enrichment and rapid detection of pathogenic bacteria based on boronate affinity systems in the future.

Although we have only studied the detection of *S. aureus* at present, the proposed strategy has shown good potential for other food pathogens considering the full development and wide application of probes specific for various bacteria [48,49,50]. Moreover, further improvements in the design and preparation of boronate affinity materials would allow more effective affinity isolation systems to be fabricated [31]. Fluorescent probe techniques are also being enhanced [51]. With the effective combination of in situ analysis techniques [52] and polymer coupling technology [53], one can expect significantly increased analytical efficiency for the established technique for fast and in situ detection of pathogens in different food samples.

## 5. Conclusions

In this study, a DFFPBA-functionalized cellulose filter paper was prepared for the detection of *S. aureus* in combination with a specific fluorescent probe. The modification with PEI was proved to significantly enhance the boronate affinity of the paper to bacteria. The fluorescent analysis demonstrated satisfactory specificity, and after optimization of bacterial enrichment and elution conditions, the LOD for *S. aureus* was calculated as 2.24 × 10^2^ CFU/mL, with the linear range of 10^4^ CFU/mL~10^8^ CFU/mL (R^2^ = 0.9835). When validated with milk and yogurt samples (spiking concentration from 10^4^ CFU/mL to 10^7^ CFU/mL), the recovery was calculated as 95.49~106.2% and the CV was 0.7~2.4%. In comparison with other methods, the established technique demonstrated excellent matrix tolerance. It could hopefully achieve more rapid, simple, sensitive, and accurate detection in food samples, which allowed us to suggest its promising potential as a new technique for effectively monitoring and controlling food pathogens.

## Figures and Tables

**Table 1:** Comparison of different fluorescent detection methods for pathogenic bacteria.

Materials and Methods	Targets	LODs	Time	Matrix Tolerance	References
Multiplex real-time PCR	*E. coli*	1.4 × 10^2^–4.3 × 10^2^ CFU/mL	6–8 h of enrichment time	Vulnerable to protein and fat and pre-enrichment required	[37]
Fluorescence in situ hybridization	*Enterobacteriaceae*/*Pseudomonas* spp.	2 × 10^3^ CFU/mL	5–7 h	Enzymatic treatment of milk samples was required to remove digested milk lipids and proteins	[38]
Electrospun boronic acid-containing polymer membranes as fluorescent sensors for bacteria detection	*E. coli*, *S. aureus* and *Pseudomonas aeruginosa*	Not reported	8–24 h	Not reported	[39]
Fluorescently labeled PAMAM with PBA; fluorescence spectrophotometry	*S. aureus* *E. coli*	10^4^ CFU/mL	30 min	Not reported	[21]
Nanosensor consisting of a diol-modified fluorescent probe and phenylboronic acid-functionalized fluorescent carbon dots	*S. aureus* *E. coli*	10 CFU/mL	Incubate for 1 h	No quantitative analysis of complex samples	[10]
Boronate affinity filter-paper-based fluorescence detection	*S. aureus*	2.24 × 10^2^ CFU/mL	3.5 h	For 10^4^ CFU/mL~10^7^ CFU/mL *S. aureus*, excellent accuracy and precision were observed	This study

## Data Availability

The data presented in this study are available on request from the corresponding author.
